# Analysing ill-conditioned Markov chains

**DOI:** 10.1098/rsta.2022.0245

**Published:** 2023-07-10

**Authors:** Esmae J. Woods, Deepti Kannan, Daniel J. Sharpe, Thomas D. Swinburne, David J. Wales

**Affiliations:** ^1^ Cavendish Laboratory, Department of Physics, University of Cambridge, Cambridge CB3 0HE, UK; ^2^ Department of Chemistry, University of Cambridge, Lensfield Road, Cambridge CB2 1EW, UK; ^3^ CNRS, CINaM UMR, Aix-Marseille Université, 7325, Campus de Luminy, 13288 Marseille, France

**Keywords:** energy landscapes, Markov chains, rare events, graph transformation, dimensionality reduction

## Abstract

Discrete state Markov chains in discrete or continuous time are widely used to model phenomena in the social, physical and life sciences. In many cases, the model can feature a large state space, with extreme differences between the fastest and slowest transition timescales. Analysis of such ill-conditioned models is often intractable with finite precision linear algebra techniques. In this contribution, we propose a solution to this problem, namely partial graph transformation, to iteratively eliminate and renormalize states, producing a low-rank Markov chain from an ill-conditioned initial model. We show that the error induced by this procedure can be minimized by retaining both the renormalized nodes that represent metastable superbasins, and those through which reactive pathways concentrate, i.e. the dividing surface in the discrete state space. This procedure typically returns a much lower rank model, where trajectories can be efficiently generated with kinetic path sampling. We apply this approach to an ill-conditioned Markov chain for a model multi-community system, measuring the accuracy by direct comparison with trajectories and transition statistics.

This article is part of a discussion meeting issue ‘Supercomputing simulations of advanced materials’.

## 1. Introduction

Complex stochastic network models are important in a wide range of fields, including socio-economics [1], epidemiology [2–4], biochemistry [5–7], systems biology [8–10], biophysics [11–15] and condensed matter [16–18]. The networks can be represented as a graph, G(V,E), where the V nodes constitute discrete microstates of the system, and the E edges are parameterized by transition rates, in the case of continuous time Markov chains (CTMCs), or by transition probabilities, in the case of discrete time Markov chains (DTMCs). In both cases, the dynamics on the network are assumed to be memoryless. The mathematical analysis of such models reveals a rich and subtle interplay between network topology and dynamics [17–23].

We are often interested in studying the transition path ensemble (TPE), namely the set of pathways from a set of initial nodes B to a set of absorbing nodes A [7,24]. However, many networks exhibit a range of dynamical time scales and when this leads to a large separation of the characteristic timescales, numerical problems become pervasive in the analysis of the corresponding Markov chains [25–29]. Because the transition matrix is ill-conditioned in a metastable Markov chain, linear algebra operations, such as eigendecomposition, for the computation of the A←B first passage time (FPT) distribution, break down due to finite precision [30]. Moreover, sampling of A←B pathways via the standard kinetic Monte Carlo (kMC) algorithm becomes unfeasible due to the ‘flickering’ of trajectories within metastable groups of nodes, which act as kinetic traps [31–34]. Dimensionality reduction aims to alleviate these problems by focusing on the slowest dynamical processes between key macrostates of interest. The resulting coarse-grained model is also easier to interpret because details of the faster processes become implicit.

A common dimensionality reduction approach is to partition the Markovian network model into communities of nodes and then estimate the rates for inter-community transitions. However, analytical methods to compute the optimal coarse-grained rate matrix for a given community structure do not scale well [35]. Simpler approximations, including the local equilibrium approximation [36], may introduce significant errors in the inter-community dynamics [35].

A time-independent rate can strictly be defined only for an inter-community transition if the FPT dynamics are ‘ideally’ metastable [30]. Here, the communities must be chosen such that the distribution of escape times from each metastable macrostate follows a single-exponential decay. However, in realistic models, it may not be possible to find such an ideal community structure, and assuming Markovianity in the reduced state space may then introduce significant errors for the inter-community dynamics [37,38]. In particular, it is especially difficult to preserve higher moments of the FPT distribution beyond the mean first passage time (MFPT). A more faithful representation of inter-community transitions is provided by the full FPT distribution from an initial macrostate B (reactants) to an absorbing macrostate A (products). However, the required eigendecomposition breaks down because of finite precision due to the inherent metastability [30]. For systems with sufficiently large spectral gaps, it is also possible to develop a formalism based on perturbation theory to describe the slower dynamical time scales [39].

An alternative approach is to employ enhanced kMC methods that leverage knowledge of the metastable macrostates to simulate non-Markovian trajectories between communities. One class of methods is based on formulating the escape of a trajectory from a metastable basin as an absorbing Markov chain, as in the Monte Carlo with absorbing Markov chains (MCAMC) [40,41] method. The key idea behind these methods is to solve the master equation for the limited number of states in a trapping basin, so that a simulated trajectory escapes the basin in a single iteration [42]. However, if the trapping basins are too large, the overhead associated with solving for the exit probabilities and FPTs to particular absorbing microstates severely limits the efficiency of the method [32,43]. A related approach is kinetic path sampling (kPS) [33,43,44], which employs graph transformation [45] (GT), a node reduction algorithm that preserves mean FPTs, and an iterative reverse randomisation procedure, to sample a stochastic escape path to the absorbing boundary of a trapping basin along with an associated waiting time. As for MCAMC, the efficiency of kPS is severely limited by the number of nodes in each metastable basin [43]. Moreover, if the communities do not appropriately characterize the metastable macrostates, simulations may become unfeasibly slow due to trajectories flickering between the community boundaries.

A promising solution to the problem of overly large communities is to eliminate ‘fast’, i.e. rapidly evolving, states that are insignificant for global dynamics. States that contribute fast dynamical eigenmodes, corresponding to unproductive fluctuations within metastable basins, stiffen the Markov chain and hamper numerical analysis [46]. If these states are removed, the fast eigenmodes are no longer present in the reduced Markov chain, thus circumventing the flickering problem that precludes the use of the standard kMC algorithm. Moreover, if the transition rate matrix of the reduced Markov chain is less ill-conditioned, linear algebra operations, such as eigendecomposition, might retain sufficient precision to compute the full FPT distribution between two endpoint macrostates in the reduced model.

The elimination of states from a Markov chain, known as state reduction [47], has previously been employed to compute dynamical quantities such as the stationary distribution [48,49], fundamental matrix [50], and MFPTs [51,52]. Similar node-elimination schemes have been used to project the master equation onto a system of slow eigenmodes [46,53–55], which has inspired several stochastic simulation algorithms for sampling rare events in stiff Markov chains [54,56,57]. These methods use state reduction as a numerical tool for extracting properties from the original Markov chain.

In this contribution, we employ GT [45] as the state reduction technique, using a recent implementation (block GT) that enables the removal of multiple states [27,34,45,58–61]. Our focus is on numerically challenging Markov chains, with predefined community structure, which cannot be analysed with numerical linear algebra techniques due to loss of numerical precision. Our main result concerns the optimal selection of states so that the reduced Markov chain retains high accuracy, as measured by the generation of statewise trajectories. We find that accurate reduced Markov chains can be obtained if we retain, not only a node to represent the minimum free energy state of each metastable community, but also ‘boundary states’ that mediate inter-community connections, and thus have maximum participation in reactive pathways. We apply our approach to a numerically challenging multi-community system, directly comparing the trajectories and transition statistics. Reduction of the network produces a lower dimension Markov chain, which can be analysed by kPS in the metastable regime.

## 2. Discrete state Markov chains

We first briefly recall some relevant properties of discrete state Markov Chains. In a discrete state space Ω, a Markov chain is completely described by a stochastic transition (branching) probability matrix B, which in continuous time, has elements $B_{ij}=K_{ij}\tau_{j}$, where $K_{ij}$ is the transition rate from j to i, and $\tau_{j}=1/\sum_{\gamma \neq j}K_{\gamma j}$ is the mean waiting time associated with node j. In matrix notation, $B=KD^{-1}$, where D is a diagonal matrix with entries $D_{ij}=\delta_{ij}/\tau_{j}$. The corresponding Markov transition matrix is Q=K−D, with the occupation probability vector P(t) for the states in Ω evolving as

2.1 $$\frac{dP(t)}{dt}=QP(t).$$

For ergodic systems (all states mutually accessible), Q has a unique equilibrium state with eigenvalue zero, right eigenvector π with components corresponding to equilibrium occupation probabilities $\pi_{i}$, and left eigenvector 1, a row vector of ones, while all the other eigenvalues are negative. It is straightforward to show that this formulation satisfies conservation of the total occupation probability, 1⋅dP(t)/dt=0. The condition Qπ=0 required for existence of a steady state is known as global balance, where π is the vector of equilibrium occupation probabilities. In the present work, we assume that the transition rates satisfy the stronger detailed balance condition, $K_{ji}\pi_{i}=K_{ij}\pi_{j}$, which maintains equilibrium at the level of individual transitions [62].

### (a) First passage time distributions

The key observable for which we will compare and evaluate the renormalized Markov chains is the *FPT distribution* into target states A∈Ω from source states B∈Ω. Consider partitioning the total space Ω into two regions Ω=A∪S, such that B∈S and A∩S=0, i.e. A and S do not overlap. Let $Q_{S}$ be the subset of the full transition matrix Q containing the inter-state transition rates within S, and let $D_{S}$ be the corresponding subset of the diagonal matrix of total rates D including the escape rates to A. As a result, the system evolving as $dP_{S}(t)/dt=Q_{S}P_{S}(t)$ will eventually decay completely, $P_{S}(t)\to 0$, because all trajectories are eventually absorbed in A for a connected network. When the detailed balance condition is satisfied, the probability distribution (density function) for A←B FPTs can be written as the sum [30]

2.2 $$p_{A\leftarrow B}(t)=\sum_{\ell =1}^{|S|}\lambda_{\ell ,S}\;e^{-\lambda_{\ell ,S}t}1_{S}(w_{\ell ,S}^{R}⊗w_{\ell ,S}^{L})P_{S}(0),$$

with the corresponding probability distribution for y=ln⁡t,

2.3 $$P_{A\leftarrow B}(y)=\sum_{\ell =1}^{|S|}\lambda_{\ell ,S}\;e^{y-\lambda_{\ell ,S}e^{y}}1_{S}(w_{\ell ,S}^{R}⊗w_{\ell ,S}^{L})P_{S}(0),$$

where $\left\{-\lambda_{\ell ,S}\right\}$ are the eigenvalues of $Q_{S}$, so that $\lambda_{\ell ,S}>0$, and $w_{\ell ,S}^{R}$ and $w_{\ell ,S}^{L}$ are the corresponding right (column) and left (row) eigenvectors. The logarithmic time distribution clearly shows distinct peaks for competing pathways [30,33,63]. The probability vector $P_{S}(0)$ has dimension |S| but the elements are non-zero only for source states in B. As $p_{A\leftarrow B}(t)$ can generate all moments of the FPT, it is an ideal observable for comparing the complete and reduced/renormalized Markov chains we treat in this paper. It will also be useful to define the fundamental matrix [64,65]

2.4 $$G_{S}=-D_{S}Q_{S}^{-1}={\left[I_{S}-B_{SS}\right]}^{-1},$$

where $I_{S}$ is the identity matrix of dimension |S| and $B_{SS}$ is the branching matrix between all pairs of states in S. The elements ${\left[G_{S}\right]}_{ss^{\prime }}$ of the fundamental, or Green’s, matrix are equal to the expected number of visits to a state s along a trajectory starting at $s^{\prime }$ and terminating anywhere at the absorbing boundary of S [62].

### (b) Metastability

This study concentrates on systems with highly metastable communities, where the expected escape time from a community is much greater than the time required to reach a local steady state. As the temperature falls, trapping basins become more stable, leading to greater metastability.

Metastability can be more precisely defined using the spectral approach introduced above. For a given set of nodes X let $Q_{X}$ be the elements of the full transition matrix Q that connect the states in X, where the diagonal matrix of total rates $D_{X}$ includes all escape rates out of X. Ordering the reversed sign eigenvalues as $0<\lambda_{0,X}\leq \lambda_{1,X}\ldots$ of $Q_{X}$, the degree of metastability is defined by the mixing time

2.5 $$\tau_{m}=\frac{1}{\left(\lambda_{1,X}-\lambda_{0,X}\right)}>0.$$

For $\lambda_{0,X}\tau_{m}\ll 1$, an initial probability density confined to X will decay to the corresponding right eigenvector $w_{0,X}^{R}$ on a timescale $\tau_{m}\ll 1/\lambda_{0,X}$, from which point the escape time distribution from X will be a simple exponential decay with an expected escape time of $1/\lambda_{0,X}$.

The distribution $w_{0,X}^{R}$ is known as the quasi-stationary distribution [66]; in the limit $\lambda_{0,X}\tau_{m}\to 0$, the quasi-stationary distribution tends to local equilibrium in X, i.e. $w_{0,X}^{R}\to \pi_{X}$, while the left eigenvector $w_{0,X}^{L}\to 1_{X}$, i.e. a row vector of ones, as discussed in previous work [61].

A consequence of metastability is that any initial distribution in X that gives negligible probability leakage from X over times $t<\tau_{m}$ will produce essentially identical FPT distributions to other communities.^1^ This relative insensitivity with respect to initial conditions will be invoked when justifying which nodes to remove in later sections.

## 3. Partial graph transformation

The GT algorithm removes nodes individually, renormalizing all retained nodes such that A←B branching probabilities and mean FPTs are unchanged [34,45,59]. This procedure was made numerically robust for arbitrarily ill-conditioned systems, by ensuring only floating point numbers of a similar magnitude are compared, making the procedure applicable for arbitrarily ill-conditioned systems [27]. The GT algorithm has recently [61] been extended for removal of multiple states simultaneously, making GT computations possible in fewer iterations [58,60,61]. When the block matrices containing all states to be removed in a single iteration are better conditioned than the entire system it may be possible to employ linear algebra methods to solve for the local dynamics. We use condition number estimations from LAPACK to revert to the numerically robust state-by-state GT algorithm when block matrices are too ill-conditioned, on-the-fly [61]. We have normally employed GT procedures to remove all states aside from sources and sinks, to compute mean FPTs. However, the FPT distribution is not available within this framework. Here, we introduce our partial GT algorithm, which retains all the boundary states between communities and the global community minima. We show that this approach can reduce the dimensionality of the network, while preserving the FPT distribution.

### (a) The partial GT procedure

We begin by partitioning the full state space Ω into a set of nodes to be eliminated Z and the set of nodes to be retained, $\Omega^{Z}$, such that $Z\cup \Omega^{Z}\equiv \Omega$. The branching probability matrix can then be written

3.1 $$B=\left[\begin{array}{cc} B_{ZZ} & B_{Z\Omega^{Z}}\\ B_{\Omega^{Z}Z} & B_{\Omega^{Z}\Omega^{Z}} \end{array}\right].$$

The renormalized branching probabilities and waiting times after removing Z with GT are given by Swinburne & Wales [61]

3.2 $$B_{\Omega^{Z}\Omega^{Z}}^{Z}=B_{\Omega^{Z}\Omega^{Z}}+B_{\Omega^{Z}Z}G_{Z}B_{Z\Omega^{Z}}$$

and

3.3 $$\tau_{\Omega^{Z}}^{Z}=\tau_{\Omega^{Z}}+\tau_{Z}G_{Z}B_{Z\Omega^{Z}},$$

where the superscript indicates the set of eliminated nodes, the subscript indicates the set of retained nodes, $G_{Z}={\left[I_{Z}-B_{ZZ}\right]}^{-1}$ is Green’s matrix for Z and $\tau_{X}=1_{X}{\text{D}}_{X}^{-1}$ is a row vector of waiting times for nodes in region X.

### (b) Properties of the renormalized Markov chain

The matrix $B_{\Omega^{Z}\Omega^{Z}}^{Z}$ describes transitions in a reduced Markov chain associated with a transition rate matrix

3.4 $${\text{Q}}_{\Omega^{Z}}^{Z}=[B_{\Omega^{Z}\Omega^{Z}}^{Z}-I_{\Omega^{Z}}]{\text{D}}_{\Omega^{Z}}^{Z},$$

where ${\left[{\text{D}}_{\Omega^{Z}}^{Z}\right]}_{ij}=\delta_{ij}/{\left[\tau_{\Omega^{Z}}^{Z}\right]}_{i}$. The matrix ${\text{Q}}_{\Omega^{Z}}^{Z}$ is less sparse than the original transition rate matrix Q because the GT procedure adds edges to the network to account for transitions via eliminated nodes (equation (3.2)).

### (c) Stationary distribution of a GT-renormalized Markov chain

For a steady state π, the *global* balance condition (existence of a steady state) requires that the total probability flux into each state is exactly balanced by the total probability flux leaving each state, leading to the equality

3.5 BDπ=Dπ.

Global balance is more general than the detailed balance condition, which imposes a flux balance for each individual state-to-state transition, giving $B_{ij}{\left[D\pi \right]}_{j}=B_{ji}{\left[D\pi \right]}_{i}$, as discussed in §2.

Consider removing a single state z through GT, giving a renormalized branching probability matrix and waiting times for the remaining states in $\Omega^{Z}$

3.6 $${\left[B_{\Omega^{Z}\Omega^{Z}}^{Z}\right]}_{ij}=B_{ij}+B_{iz}B_{zj}/(1-B_{zz})$$

and

3.7 $${\left[\tau_{\Omega^{Z}}^{Z}\right]}_{i}=\tau_{i}+\tau_{z}B_{zi}/(1-B_{zz}).$$

It is straightforward to show that the vector ${\left[f\right]}_{i}={\left[D\pi \right]}_{i}$, with i≠z, satisfies the new global balance condition

$$\begin{array}{rl} \sum_{\omega \in \Omega^{Z}}{\left[B_{\Omega^{Z}\Omega^{Z}}^{Z}\right]}_{i\omega }f_{\omega } & \;={\left[BD\pi \right]}_{i}-B_{iz}{\left[D\pi \right]}_{z}+\frac{B_{iz}}{1-B_{zz}}({\left[BD\pi \right]}_{z}-B_{zz}{\left[D\pi \right]}_{z})\\ & \;=f_{i}+\frac{B_{iz}}{1-B_{zz}}({\left[D\pi \right]}_{z}-B_{zz}{\left[D\pi \right]}_{z}-{\left[D\pi \right]}_{z}+B_{zz}{\left[D\pi \right]}_{z})=f_{i}. \end{array}$$

We require the new steady-state distribution $\pi_{\Omega^{Z}}^{Z}$ to satisfy $B_{\Omega^{Z}\Omega^{Z}}^{Z}D_{\Omega^{Z}}^{Z}\pi_{\Omega^{Z}}^{Z}=D_{\Omega^{Z}}^{Z}\pi_{\Omega^{Z}}^{Z}$. Since $B_{\Omega^{Z}\Omega^{Z}}^{Z}f=f$ it follows that ${\left[D_{\Omega^{Z}}^{Z}\pi_{\Omega^{Z}}^{Z}\right]}_{i}\propto {\left[D\pi \right]}_{i}$ for $i\in \Omega^{Z}$. Normalization of π over Ω and $\pi_{\Omega^{Z}}^{Z}$ over $\Omega^{Z}$ shows that the constant of proportionality is unity. Hence

3.8 $$\frac{{\left[\pi_{\Omega^{Z}}^{Z}\right]}_{i}}{{\left[\tau_{\Omega^{Z}}^{Z}\right]}_{i}}=\frac{\pi_{i}}{\tau_{i}}\;\mathrm{or}\;\frac{\pi_{i}^{Z}}{\pi_{i}}=\frac{\tau_{i}^{Z}}{\tau_{i}}\;\mathrm{for}\;i\in \Omega^{Z}\;\mathrm{with}\;\pi_{i}^{Z}\equiv {\left[\pi_{\Omega^{Z}}^{Z}\right]}_{i},\;\tau_{i}^{Z}\equiv {\left[\tau_{\Omega^{Z}}^{Z}\right]}_{i}.$$

As this result generalizes to the GT removal of multiple states, we see that for each individual state $i\in \Omega^{Z}$ the renormalized steady-state occupation probabilities are *not* proportional to the original equilibrium occupation probabilities of the retained nodes, but reweights each retained state $i\in \Omega^{Z}$ by the factor $\tau_{i}^{Z}/\tau_{i}$ involving the renormalized and original waiting times.

In summary: if the original rate matrix satisfies detailed balance with distribution π, the renormalized rate matrix ${\text{Q}}_{\Omega^{Z}}^{Z}$ satisfies detailed balance with distribution $\pi_{\Omega^{Z}}^{Z}$.

## 4. Dimensionality reduction of networks with partial GT

In this section, we use the partial GT procedure outlined above to coarse-grain a general Markov chain with a predefined community structure, which maps any state i∈Ω to non-overlapping communities {A,B,…} through some participation function

4.1 C(i)∈{A,B,…},i∈Ω.

Any coarse-graining procedure inevitably leads to information loss, incurring error. In the present case, this error can be precisely defined in certain limits, as GT exactly preserves the branching probabilities, $B_{ij}$, and MFPT between retained nodes. This result also applies to inter-community MFPTs, using (2.2),

4.2 $$T_{AB}\equiv \int_{0}^{\infty }t\;p_{A\leftarrow B}(t)\;dt,$$

which additionally requires specification of some initial distribution for the first passage process. Providing the initial distribution in B is only over retained nodes, GT also preserves $T_{AB}$ [61]. For metastable communities, a plausible choice is the local equilibrium distribution $\pi_{B}$ of the initial community.

### (a) Boundary nodes

When considering which nodes to remove with GT, giving reduced communities $\left\{A^{Z},B^{Z},\ldots \right\}$, it is important to note that $T_{AB}$ measures the average time to reach the *boundary nodes* ∂A of the target community A. The boundary nodes are defined as the nodes that have direct connections to multiple communities. This observation allows us to write

4.3 $$T_{AB}\equiv T_{\partial AB},$$

where the initial distribution in B is still unspecified. When building a reduced Markov model, we must consider A←B and B←A processes. To preserve MFPTs between reduced communities, and therefore maintain accuracy in the FPT distributions, we retain the boundary nodes of all communities, i.e. we require $\partial X=\partial X^{Z},X=A,B,\ldots$.

### (b) Initial distributions under metastability

Section 2(b) discussed the metastable limit, where the mixing timescale $\tau_{m}$ in equation (2.5) of some community is much smaller than the characteristic escape time from that community. It is important to note that for systems with more than two communities, the escape and first passage times are quite distinct quantities. In the metastable limit, any initial distribution that has negligible probability of escape over times $t<\tau_{m}$ will have essentially identical first passage statistics.

A plausible choice of initial distribution for the first passage calculation (4.2) is the local equilibrium distribution $\pi_{B}$, which for metastable communities will typically have negligible probability of escape over times $t<\tau_{m}$. However, the insensitivity to initial conditions allows us to instead take an initial distribution concentrated entirely on some interior community state, such as the one with the minimal free energy.

This choice motivates a reduced state space for a community B of

4.4 $$B^{Z}\equiv \partial B\cup b_{0},\;b_{0}=\arg\max_{b\in B}\pi_{b},$$

such that the boundary nodes remain, i.e. $\partial B^{Z}=\partial B$, which ensures that the reduced state space also preserves the reverse MFPT, $T_{AB}$.

By taking the same initial distribution, concentrated on $b_{0}$, for the first passage calculation (4.2) in the full and reduced network, we can ensure that the MFPT between communities is exactly preserved. This property can be written as $T_{A^{Z}B^{Z}}=T_{A^{Z}B^{Z}}^{Z}$, i.e. the MFPT from $B^{Z}$ to $A^{Z}$ is unchanged whether calculated for the full or GT reduced model.

Although we find that retaining boundary nodes gives an accurate and much lower rank model in the metastable limit, the model is not necessarily better conditioned since both fast and slow rates are retained. Nevertheless, the reduced rank significantly aids interpretation and renders kPS methods [33,43,44], which employ GT to overcome ill-conditioning problems, highly efficient.

## 5. Application to a model nine-community network

We now apply partial GT to reduce the number of nodes within each metastable community in a transition network, resulting in a coarse-grained model that can be more easily analysed with enhanced kMC methods, such as kPS, which scale poorly with community size.

**Figure 1.**
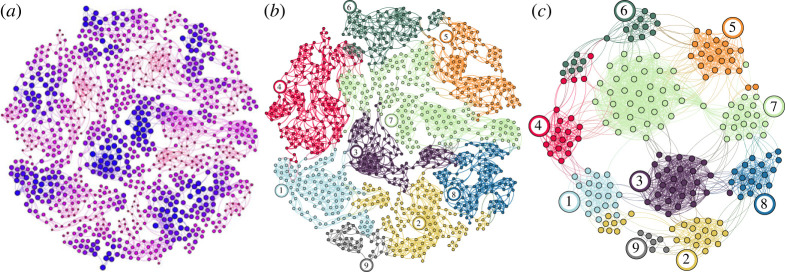
Dimensionality reduction pipeline for a model network (visualized using Gephi [67]) consisting of 994 nodes and 4320 bidirectional edges, parameterized by Arrhenius transition rates. (*a*) The network is embedded in a two-dimensional nine-well potential, which is clear in the disconnectivity graph shown in figure 2*a*. Larger darker blue nodes are associated with lower energies (higher equilibrium probabilities) and smaller darker red nodes are associated with higher energies (lower stationary probabilities). (*b*) First, the network is partitioned into nine metastable communities (communities of nodes), corresponding to the nine potential energy basins, using the Bayesian agglomerative clustering engine (BACE). (*c*) Partial GT is used to iteratively eliminate all but the lowest-energy node internal to each community, and the boundary nodes that connect communities, resulting in a reduced network with 215 nodes and 2217 edges. This reduction ensures all dominant basin escape paths from each community are preserved in a renormalized form. (Online version in colour.)

We consider a model kinetic transition network with 994 nodes and 4320 bidirectional edges embedded in a two-dimensional potential energy surface with nine principal communities, illustrated in figure 1. The network is designed to mimic the energy landscape of a molecular system, with a Poissonian node degree distribution [68] and multi-pathway kinetics for transitions between competing communities [69]. The community participation is determined using the Bayesian agglomerative clustering engine (BACE) [70], as implemented in the PyEmma [71] software package. The time scale for inter-community MFPTs ranges from $10^{3}$ at temperature T=10, to $10^{12}$ at T=1. It is useful to compare these values with the typical mean waiting times of $10^{0}$ at T=10 and $10^{2}$ at T=1, and mixing times $\tau_{m}$, of around $10^{1}$ at T=10 and $10^{3}$ at T=1. Thus, inter-community transitions are ‘rare events’ at lower temperatures, although the entire model remains sufficiently well-conditioned at T=1 to be treatable with standard linear algebra routines, allowing us to generate essentially exact reference data.

**Figure 2.**
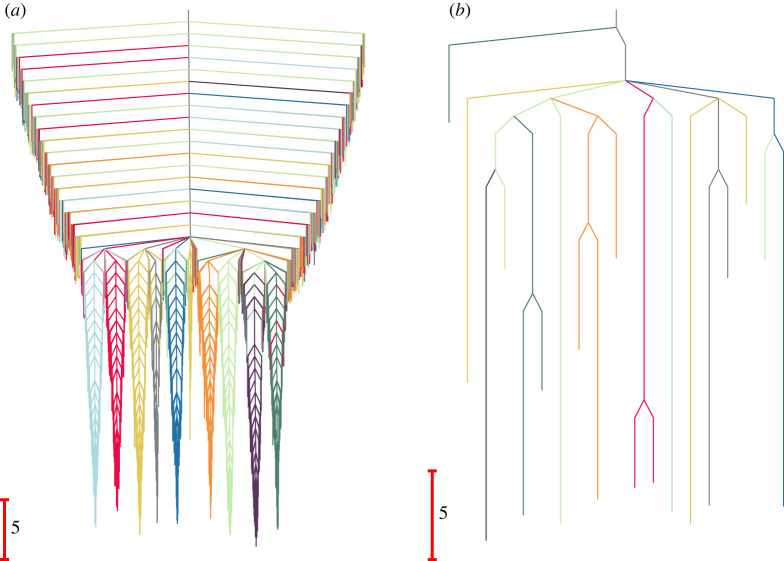
Disconnectivity graphs [72,73] for the nine-community model network shown in figure 1*a*. (*a*) The landscape for the complete network with 994 local minima. (*b*) The landscape including only minima that correspond to monotonic sequence basins, defined by minima that are not directly connected to any lower energy neighbour [74–78]. The colour scheme highlights the different communities, as labelled in figure 1. (Online version in colour.)

The organization of the landscape is clearly visible in the disconnectivity graph [72,73] shown in figure 2*a*. A further disconnectivity graph is generated by exploiting the concept of monotonic sequences, where the energy of the local minima decreases at every step [75–78]. If we include only the termini of such sequences then we restrict the disconnectivity graph to minima that have no directly connected minimum of lower energy [74]. The resulting graph highlights the monotonic sequence basins, as shown in figure 2*b*.

Following the procedure detailed in §4, a reduced network was then produced with partial GT, at T=1 and T=10, retaining boundary nodes and the minimum free energy node for each community. The resulting renormalized network, visualized in figure 1*c*, contains only 215 nodes and 2217 edges, representing an 80% reduction in the state space. The 215-node network cannot be reduced further without eliminating the boundary nodes and therefore represents the maximum level of dimensionality reduction possible without incurring significant error in the inter-community MFPTs.

**Figure 3.**
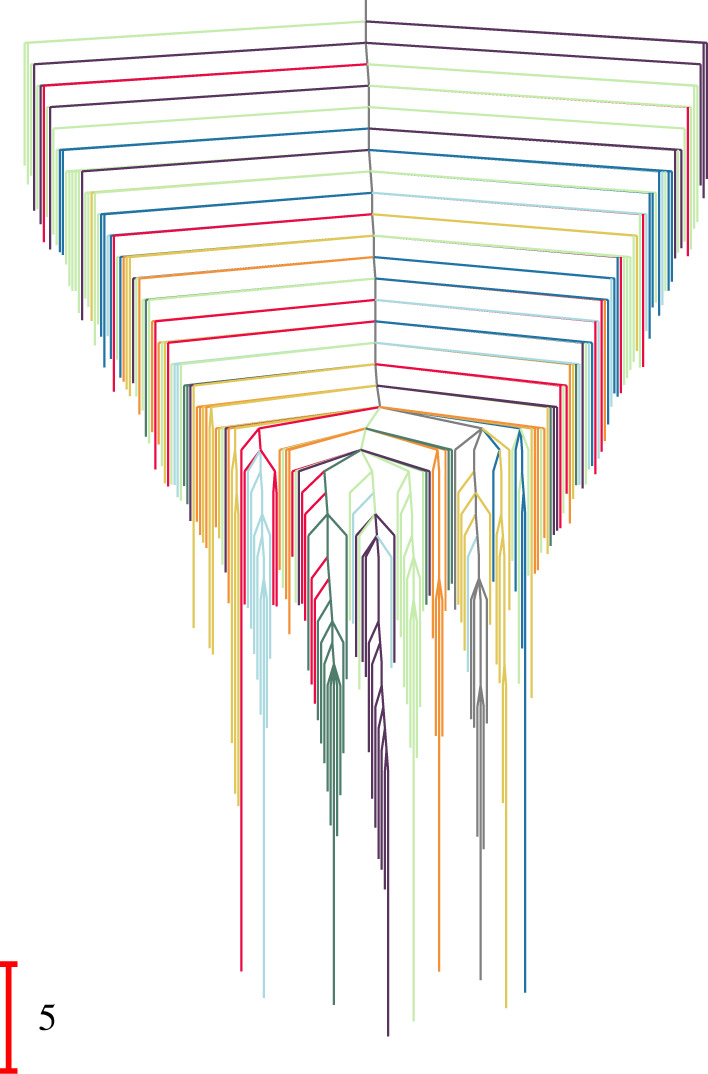
Disconnectivity graph [72,73] for the network in figure 1*c*, where 215 states are retained using the partial GT approach. This graph is based on effective free energies at T=1, as described in the text. (Online version in colour.)

The renormalized equilibrium probabilities, $\pi_{\Omega^{Z}}^{Z}$, and branching probabilities, $B_{\Omega^{Z}\Omega^{Z}}^{Z}$, can be used to construct a disconnectivity graph for the reduced network produced using the partial GT procedure (figure 3). We define effective free energies for the retained minima, $f_{\omega }(T)$, and the transition states that connect them, $f_{\omega \omega^{\prime }}^{†}(T)$, using

$$f_{\omega }(T)=-k_{B}T\ln({\left[\pi_{\Omega^{Z}}^{Z}\right]}_{\omega })$$

and

$$\begin{array}{rl} f_{\omega \omega^{\prime }}^{†}(T) & \;=f_{\omega^{\prime }}(T)-k_{B}T\ln(\frac{{\left[B_{\Omega^{Z}\Omega^{Z}}^{Z}\right]}_{\omega \omega^{\prime }}}{{\left[\tau_{\Omega^{Z}}^{Z}\right]}_{\omega^{\prime }}})+k_{B}T\ln\left(\frac{k_{B}T}{h}\right),\\ & \;=f_{\omega }(T)-k_{B}T\ln(\frac{{\left[B_{\Omega^{Z}\Omega^{Z}}^{Z}\right]}_{\omega^{\prime }\omega }}{{\left[\tau_{\Omega^{Z}}^{Z}\right]}_{\omega }})+k_{B}T\ln\left(\frac{k_{B}T}{h}\right), \end{array}$$

where the Boltzmann and Planck constants, $k_{B}$ and h, are set to unity in the reduced unit system. The ratio ${\left[B_{\Omega^{Z}\Omega^{Z}}^{Z}\right]}_{\omega^{\prime }\omega }/{\left[\tau_{\Omega^{Z}}^{Z}\right]}_{\omega }$ corresponds to the rate constant ${\left[K_{\Omega^{Z}\Omega^{Z}}^{Z}\right]}_{\omega^{\prime }\omega }$ in the new network. Hence, the free energy of the transition state is defined to reproduce the rate as

5.1 $${\left[K_{\Omega^{Z}\Omega^{Z}}^{Z}\right]}_{\omega^{\prime }\omega }=\frac{k_{B}T}{h}\exp\left[-\frac{\left(f_{\omega \omega^{\prime }}^{†}(T)-f_{\omega }(T)\right)}{k_{B}T}\right].$$

### (a) First escape time distributions

First escape time distributions were calculated for each community, with the system initialized in different starting configurations, using eigendecomposition of the probability matrix, for the full network. We look at four different starting distributions for the full network: (a) Boltz: probability is distributed according to the local Boltzmann distribution within the starting community; (b) Min: probability is localized in the global minimum free energy state of the starting community; (c) Uni: probability is uniform over all states in the community; and (d) Mix: probability is initially uniform, then conditioned to remain within the starting community for a mixing time $\tau_{m}$.

Cases (a) and (b) are plausible initial distributions for first escape and first passage problems, while the uniform distributions (c) and (d) are designed to test the sensitivity to initial conditions, rather than representing any physical scenario.

The distribution in (d) can be produced using the eigenvalues and eigenvectors of the rate matrix for some starting community X, as discussed in (b). The initially uniform density in X is first evolved to time $\tau_{m}$ via

5.2 $$p_{X}(\tau_{m})=\sum_{l}\exp(-\tau_{m}\lambda_{l,X})w_{l,X}^{R}w_{l,X}^{L}p_{X}(0).$$

The resulting distribution is then normalized and used as the initial distribution for (2.2), giving a first escape time distribution shifted by $\tau_{m}$. Access to the full eigenspectrum of a local community requires stability of linear algebra routines, which limits the range of temperatures at which this artificial test distribution can be evaluated.

**Figure 4.**
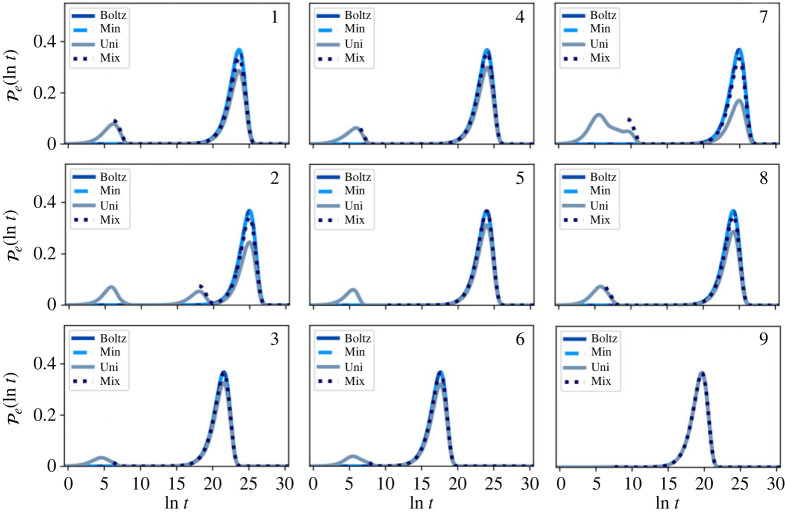
First escape time distributions for the nine-community model, before partial GT at T=1. The system is initialized in four different starting configurations, Boltz, Min, Uni and Mix, within each community, as labelled in the top right corner of each plot. An additional small peak is seen at small time for Uni, due to significant starting probability in boundary nodes causing rapid escape. At this low temperature, most of the probability in the initial Boltzmann distribution is localized in or near the global minimum of the community, which results in almost identical escape distributions for Boltz and Min. For longer times, Mix tends towards the Boltzmann distribution. (Online version in colour.)

**Figure 5.**
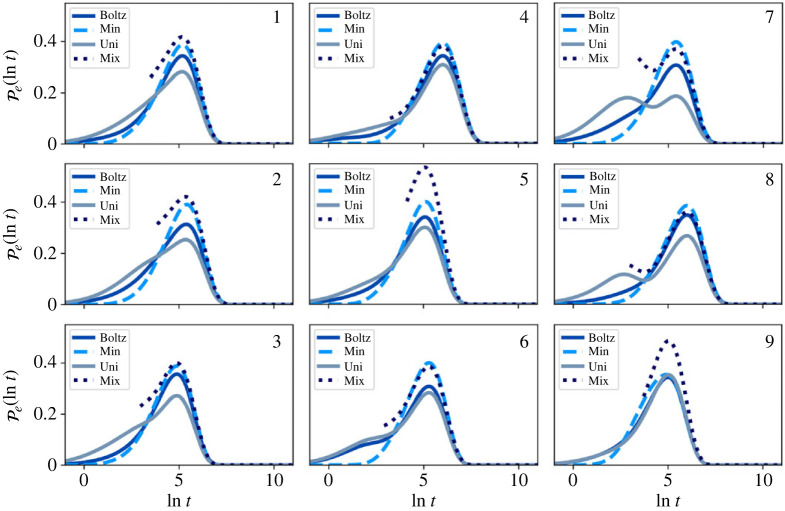
First escape time distributions for the nine-community model, before partial GT at T=10. The system is initialized in four different starting configurations, Boltz, Min, Uni and Mix, within each community, as labelled in the top right corner of each plot. Both the Boltz and Uni escape distributions have larger escape probabilities at small time compared with Min, due to significant starting probabilities in boundary nodes. Mix shows a peak centred on the Min peak, but of different height, as both curves are normalized to have unit area. (Online version in colour.)

The resulting distributions for T=1 and T=10, for the full network (before partial GT), are shown in figures 4 and 5. As discussed in the previous section, any normalized starting distribution in the metastable limit will quickly decay to the quasi-stationary distribution, which itself will be close to local equilibrium $\pi_{B}$ or $\pi_{B}^{Z}$. This result implies that the escape statistics will be essentially invariant to changes in the initial distribution for times longer than some short mixing time.

At low temperature, we see that (a), (b) and (d) all give very similar first passage distributions. For the local Boltzmann distribution at T=1, the majority of the probability (25–99%) is localized in the lowest minimum of the starting community, while at T=10 this probability drops to around 3%. For T=1, we see that the (a) and (b) escape distributions are indistinguishable.

Case (c) causes some initial probability to quickly leak to neighbouring communities due to significant starting probabilities on boundary nodes. At T=1, this effect causes a distinct peak at short time, while at T=10, the short time and long time peaks overlap, producing one strongly asymmetrical peak, with larger probability at short time. However, when we restrict to a uniform initial configuration, conditional on not leaving the starting community on timescale $t<\tau_{m}$, i.e. case (d), the resulting escape distribution moves towards the local Boltzmann distribution, illustrating the limit on invariance to initial conditions.

### (b) First passage time distributions

FPT distributions were also calculated for every community pairing for the full network, before partial GT, with the system initialized in the same four starting configurations: (a) Boltz, (b) Min, (c) Uni and (d) Mix.

**Figure 6.**
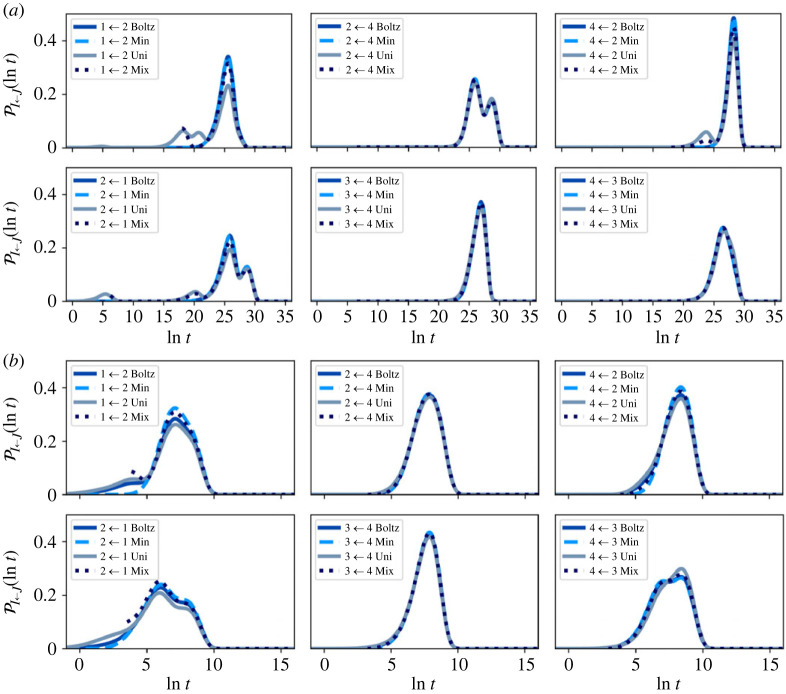
Representative first passage time (FPT) distributions for the full nine-community model before partial-GT at (*a*) T=1 and (*b*) T=10. The system is initialized in four different starting configurations, within each community: Boltz, Min, Uni and Mix. The FPT results are rather similar for the different initial conditions. Starting in a uniform distribution can produce additional peaks in the FPT distribution, but these peaks become less prominent for Mix. (Online version in colour.)

A representative selection of FPT distributions calculated using eigendecomposition is shown in figure 6. The distribution shape is not the single peak expected for a two state network, because reactive trajectories spend significant amounts of time in communities other than the reactant and product. These inter-community dynamics are controlled by the boundary nodes, which establishes the importance of these states in the retained network.

As was the case for the escape time distributions, the FPT distributions are very similar for different initial conditions. When the system is initialized in a uniform distribution, the FPT distribution can include distinct additional lower time peaks at T=1, as seen for the 2←1 transition. Additional peaks occur at very short times when the source and sink communities are neighbours. Even when communities are not neighbours, additional peaks can be seen if significant probability quickly transfers to another community (i.e. on a time scale less than the mixing time), as the resulting FPT distribution will also pick up the FPT from this community to the sink. This additional peak may be at a longer or shorter time than the main peak. Once again, initial condition (d) tends towards initial condition (a), confirming the insensitivity to initial distributions.

### (c) Validation of the partial GT reduced network

To benchmark the effectiveness of partial GT reduction, we compare first escape time distributions, FPT distributions and simulation trajectories between the full and GT-reduced networks.

**Figure 7.**
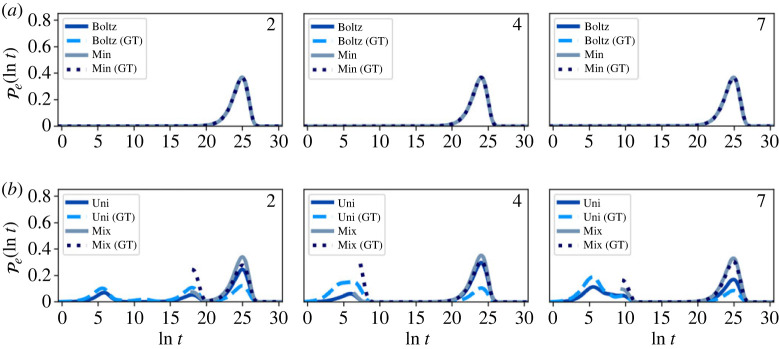
First escape time distributions for transitions between different communities in the nine-community model at T=1 for both the full and graph transformed (GT) networks, calculated using eigendecomposition. The system is initialized in different local distributions, (*a*) Boltz and Min, (*b*) Uni and Mix, within the starting communities, which are labelled in the top right corner of each panel. (Online version in colour.)

**Figure 8.**
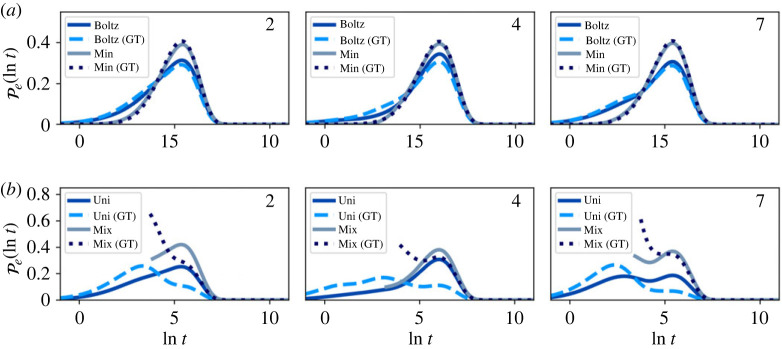
First escape time distributions for transitions between different communities in the nine-community model at T=10 for both the full and graph transformed (GT) networks, calculated using eigendecomposition. The system is initialized in different local distributions, (*a*) Boltz and Min, (*b*) Uni and Mix, within the starting communities, which are labelled in the top right corner of each panel. (Online version in colour.)

**Figure 9.**
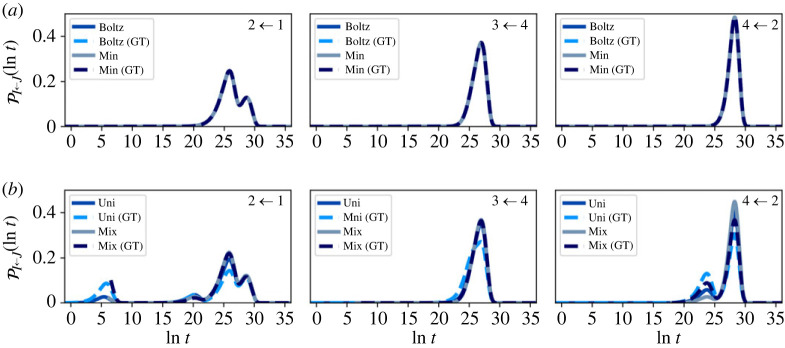
First passage time (FPT) distributions for transitions between different communities in the nine-community model at T=1 for both the full and graph transformed (GT) networks, calculated using eigendecomposition. The system is initialized in different local distributions within the starting communities: (*a*) Boltz and Min, (*b*) Uni and Mix. (Online version in colour.)

**Figure 10.**
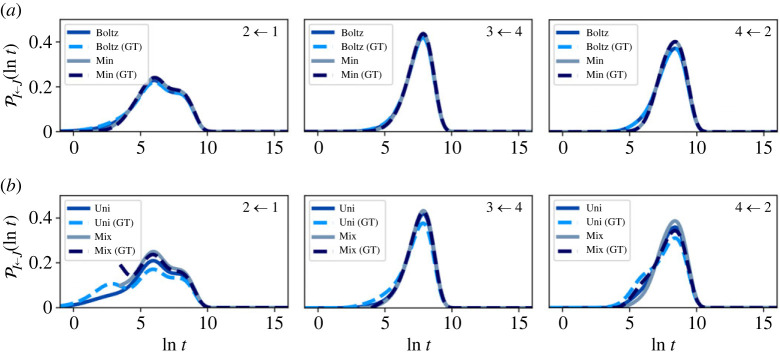
First passage time distributions for transitions between different communities in the nine-community model at T=10 for both the full and graph transformed (GT) networks, calculated using eigendecomposition. The system is initialized in different local distributions within the starting communities: (*a*) Boltz and Min, (*b*) Uni and Mix. (Online version in colour.)

Representative first escape and passage time distributions comparing the full and partial GT reduced network are shown in figures 7–10. At T=1, the reduced and full networks have identical distributions when initialized in Boltz or Min, showing that partial GT can preserve higher order moments of the FPT. Starting from a uniform distribution within the reduced network more strongly perturbs the distributions compared with the full network, due to the significant proportion of probability in the boundary nodes. However, if the system is initialized such that no probability leaves on a time scale less than the mixing time, the full and reduced distributions match quite well. Since the system becomes less metastable as the temperature increases, the matching between full and reduced networks decreases. At these higher temperatures, the Boltzmann distribution becomes more uniform, which results in better agreement across all initial conditions. Overall, the accuracy of the GT reduced network is clear. Only at the higher temperature T=10, where metastability is reduced, is some appreciable disagreement seen between full and GT reduced, for initial conditions, Boltz and Min.

As a final benchmark, we simulated 1000 non-equilibrium trajectories using kPS, initialized according to the local Boltzmann distribution of each of the nine communities of the original network, for a fixed trajectory time, at T=1. We also simulated another set of trajectories, for the same elapsed time, on the maximally GT reduced 215-node network. Three repeats were run for each system.

**Figure 11.**
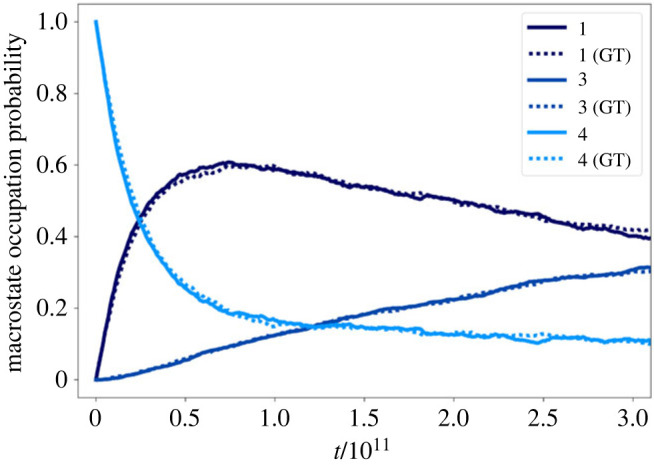
Community occupation probability, defined as the fraction of simulated trajectories assigned to a given community, as a function of simulation time. A set of 1000 trajectories initialized according to the local equilibrium distribution in community 4 of the nine-community model network (as shown in figure 1*b*) were simulated using kPS at a temperature T=1. The time-dependent occupation probabilities derived from trajectories on the original network closely match those derived from simulated trajectories on the GT-reduced 215-node network (figure 1*c*), which only retains the boundary nodes and the internal node with the largest stationary probability in each community. Since community 4 is metastable, the evolution of its occupation probability closely matches a simple exponential decay. Over time, the probability flow leaks into neighbouring communities, as shown by the gradual increase in the occupation probabilities of the neighbouring communities 3 and 1. The dynamics are accurately represented even at longer time scales, i.e. at 10 times the mean escape time from community 4. (Online version in colour.)

Figure 11 shows the time-dependent community occupation probabilities obtained from three sets of 1000 trajectories initialized in community 4 of the network in figure 1*b*. These community occupation probabilities closely follow those derived from the 1000 trajectories initialized in community 4 of the reduced network in figure 1*c*. Thus, trajectories simulated on GT-reduced networks successfully reproduce the dynamical properties of the original model.

Although partial GT significantly reduces the number of nodes in each community, it also decreases the sparsity of the network, which can hinder the efficiency of kPS [43]. Nonetheless, the CPU time required to simulate 9000 trajectories on the reduced network represents a significant speedup compared with the CPU time required for simulations on the original network (table 1).

**Table 1.** Wall clock time (ran on all 16 processors of an eight dual-core Intel(R) Core(TM) i7-11700 2.50GHz) for simulating nine sets of 1000 trajectories initialized from each of the nine communities of the original and reduced versions of the model network in figure 1.

wall clock time in original network	wall clock time in reduced network
729 min	21 min

The trade-off between dimensionality and sparsity does not affect the time complexity of MCAMC, which relies on eigendecomposition of the transition matrix for each community subnetwork to compute basin escape statistics [40,41]. Therefore, dimensionality reduction with partial GT should provide an even greater simulation speedup for MCAMC.

### (d) First passage time distributions in the metastable regime

Although inter-community FPT distributions are well preserved in GT-reduced networks, calculation of these distributions using linear algebra routines fails for ill-conditioned problems. Although we can always extract MFPTs through application of GT, trajectory and FPT distribution information provides more insight into non-equilibrium dynamics [43]. These distributions can be obtained efficiently using enhanced kMC methods that rely on a partitioning of state space, such as kPS [33,43,44] and MCAMC [40,41].

In practice, the cubic scaling of MCAMC and kPS methods with community size presents problems for application to realistic systems [33,40,43]. This issue motivates the use of partial GT to subsume fast internal nodes, to improve scalability without introducing significant errors.

**Figure 12.**
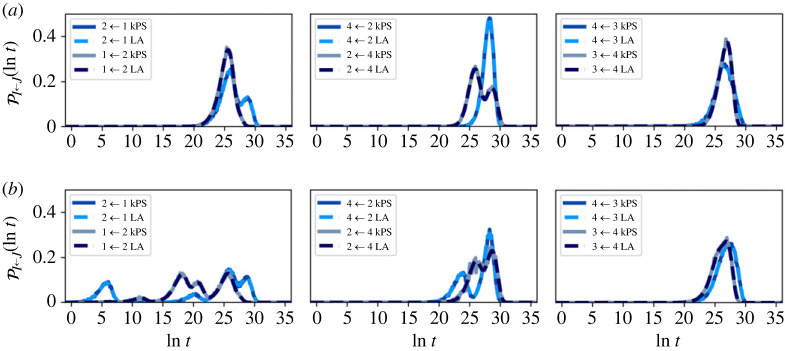
First passage time (FPT) distributions for transitions between different communities in the nine-community model at T=1. Distributions were computed using linear algebra eigendecomposition methods (LA) and kinetic path sampling (kPS) on the partial-GT reduced network. Excellent agreement is obtained between the two methods, for the different initial starting distributions of (*a*) stationary, (*b*) uniform across all states. (Online version in colour.)

**Figure 13.**
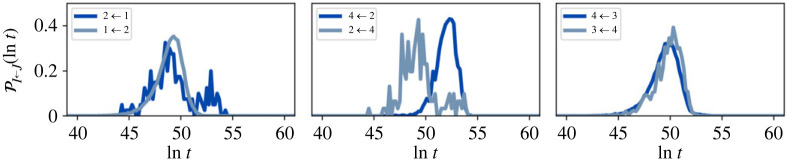
Low temperature first passage time (FPT) distributions for transitions between different communities in the nine-community model at T=0.5, computed using kinetic path sampling (kPS) on the reduced network. At this temperature, eigendecomposition is unable to produce the FPTs due to the increase of metastability causing loss of precision. However, kinetic path sampling remains functional, and due to the lower dimension of the reduced partial-GT network, is computationally feasible. The system is initialized according to the local equilibrium distribution within the starting community. At this low temperature, the efficiency of kPS varies between pairings, with some of the more difficult pairings being the passage times to community 2. (Online version in colour.)

First, we compare FPT distributions computed using kPS and linear algebra techniques, in figure 12, which shows excellent agreement. Just below T=0.8 and T=0.7, for the full and reduced system, respectively, the system becomes too ill-conditioned for eigendecomposition to succeed, and we must reply on methods which are immune to high metastability, such as kPS. At the lower temperature of T=0.5, kPS on the full network is unfeasible due to a large computational time. However, on the reduced network, kPS remains viable and FPTs can be computed, as in figure 13.

## 6. Conclusion

In this paper, we have investigated computationally stable and efficient techniques to study the flow of probability in numerically challenging Markov chains, where standard linear algebra techniques fail due to loss of numerical precision. Our main result is that the state reduction technique GT [45], in particular, a recent extension that enables the removal of multiple states [27,34,45,58–61], can produce a reduced network of much smaller size, while retaining highly accurate kinetics compared with the full network, as measured through FPT distributions. The optimal node removal strategy retains only the boundary states connecting multiple communities and the minimum free energy state within each community. This approach can be justified theoretically by considering the kinetics of first passage processes and their relative insensitivity to initial conditions in the metastable regime.

While the reduced network is much smaller, the retention of boundary states, essential for kinetic accuracy, means ill-conditioning issues still remain. However, the small network size renders numerically stable kPS techniques more efficient, providing access to the full FPT distribution.

The FPT distribution gives a much richer insight into system kinetics than MFPT calculations [63], which obscure the presence of multiple distinct relaxation time scales. The present results highlight the importance of such effects for complex, multi-community systems, which likely play a key role in multi-functional systems with multi-funnel energy landscapes [11,63]. The present contribution shows that these dynamical signatures can be captured even for numerically challenging systems, which cannot be treated with standard approaches.

## Data Availability

The graph transformation and dimensionality reduction analysis used the freely available PyGT python package [79], with additional calculations using PATHSAMPLE [80], while the kinetic path sampling simulations presented were performed with DISCOTRESS simulation Suite [81]. The nine-community dataset used in this study is freely available online [82].

## Footnotes

1 It can be shown that the error is proportional to $\exp(-t/\tau_{m})$ [66].

## References

[1] Kenett DY, Havlin S. 2015 Network science: a useful tool in economics and finance. Mind Soc. **14**, 155-167. (10.1007/s11299-015-0167-y)

[2] Simon PL, Taylor M, Kiss IZ. 2011 Exact epidemic models on graphs using graph-automorphism driven lumping. J. Math. Biol. **62**, 479-508. (10.1007/s00285-010-0344-x) 20425114 PMC7079990

[3] Pastor-Satorras R, Castellano C, Van Mieghem P, Vespignani A. 2015 Epidemic processes in complex networks. Rev. Mod. Phys. **87**, 925. (10.1103/RevModPhys.87.925)

[4] Goltsev AV, Dorogovtsev SN, Oliveira JG, Mendes JFF. 2012 Localization and spreading of diseases in complex networks. Phys. Rev. Lett. **109**, 128702. (10.1103/PhysRevLett.109.128702) 23006000

[5] Anderson DF, Kurtz TG. 2011 Continuous time Markov chain models for chemical reaction networks. In *Design and analysis of biomolecular circuits*, pp. 3–42. New York, NY: Springer.

[6] Li X, Kolomeisky AB. 2013 Mechanisms and topology determination of complex chemical and biological network systems from first-passage theoretical approach. J. Chem. Phys. **139**, 144106. (10.1063/1.4824392) 24116602 PMC3808428

[7] Allen RJ, Warren PB, Rein Ten Wolde P. 2005 Sampling rare switching events in biochemical networks. Phys. Rev. Lett. **94**, 018104. (10.1103/PhysRevLett.94.018104) 15698138

[8] Price ND, Shmulevich I. 2007 Biochemical and statistical network models for systems biology. Curr. Opin. Biotech. **18**, 365-370. (10.1016/j.copbio.2007.07.009) 17681779 PMC2034526

[9] Nakao H, Mikhailov AS. 2010 Turing patterns in network-organized activator-inhibitor systems. Nat. Phys. **6**, 544-550. (10.1038/nphys1651)

[10] Wang G, Zaman MH. 2010 Communications: Hamiltonian regulated cell signaling network. J. Chem. Phys. **132**, 121103. (10.1063/1.3357980) 20370106 PMC2859080

[11] Röder K, Joseph JA, Husic BE, Wales DJ. 2019 Energy landscapes for proteins: from single funnels to multifunctional systems. Adv. Theory Simul. **2**, 1800175.

[12] Zhou H, Wang F, Bennett DIG, Tao P. 2019 Directed kinetic transition network model. J. Chem. Phys. **151**, 144112. (10.1063/1.5110896) 31615261 PMC6800283

[13] Husic BE, Pande VS. 2018 Markov state models: from an art to a science. J. Am. Chem. Soc. **140**, 2386-2396. (10.1021/jacs.7b12191) 29323881

[14] Prinz J-H, Wu H, Sarich M, Keller B, Senne M, Held M, Chodera JD, Schütte C, Noé F. 2011 Markov models of molecular kinetics: generation and validation. J. Chem. Phys. **134**, 174105. (10.1063/1.3565032) 21548671

[15] Noé F, Rosta E. 2019 Markov models of molecular kinetics. J. Chem. Phys. **151**, 190401. 31757166 10.1063/1.5134029

[16] Cameron M, Vanden-Eijnden E. 2014 Flows in complex networks: theory, algorithms, and application to Lennard-Jones cluster rearrangement. J. Stat. Phys. **156**, 427-454. (10.1007/s10955-014-0997-8)

[17] Doye JPK, Massen CP. 2005 Characterizing the network topology of the energy landscapes of atomic clusters. J. Chem. Phys. **122**, 084105. (10.1063/1.1850468) 15836018

[18] Doye JPK. 2002 Network topology of a potential energy landscape: a static scale-free network. Phys. Rev. Lett. **88**, 238701. (10.1103/PhysRevLett.88.238701) 12059405

[19] Newman MEJ. 2009 Networks. Oxford, UK: Oxford University Press.

[20] Porter M, Gleeson J. 2016 *Dynamical systems on networks*, vol. 4 of *Frontiers in Applied Dynamical Systems: Reviews and Tutorials*. Cham, Switzerland: Springer International Publishing.

[21] Barzel B, Barabási A-L. 2013 Universality in network dynamics. Nat. Phys. **9**, 673-681. (10.1038/nphys2741) 24319492 PMC3852675

[22] Harush U, Barzel B. 2017 Dynamic patterns of information flow in complex networks. Nat. Commun. **8**, 2181. (10.1038/s41467-017-01916-3) 29259160 PMC5736766

[23] Shirley MDF, Rushton SP. 2005 The impacts of network topology on disease spread. Ecol. Complex. **2**, 287-299. (10.1016/j.ecocom.2005.04.005)

[24] Allen RJ, Frenkel D, Wolde PRt.. 2006 Simulating rare events in equilibrium or nonequilibrium stochastic systems. J. Chem. Phys. **124**, 024102. (10.1063/1.2140273) 16422566

[25] Nagahata Y, Maeda S, Teramoto H, Horiyama T, Taketsugu T, Komatsuzaki T. 2016 Deciphering time scale hierarchy in reaction networks. J. Phys. Chem. B **120**, 1961-1971. (10.1021/acs.jpcb.5b09941) 26641663

[26] Frankcombe TJ, Smith SC. 2009 Numerical solution methods for large, difficult kinetic master equations. Theor. Chem. Acc. **124**, 303-317. (10.1007/s00214-009-0623-z)

[27] Stevenson JD, Wales DJ. 2014 Communication: analysing kinetic transition networks for rare events. J. Chem. Phys. **141**, 041104. (10.1063/1.4891356) 25084870

[28] Philippe B, Saad Y, Stewart WJ. 1992 Numerical methods in Markov chain modeling. Oper. Res. **40**, 1156-1179. (10.1287/opre.40.6.1156)

[29] Meyer CD Jr. 1994 Sensitivity of the stationary distribution of a Markov chain. SIAM J. Matrix Anal. Appl. **15**, 715-728.

[30] Swinburne TD, Kannan D, Sharpe DJ, Wales DJ. 2020 Rare events and first passage time statistics from the energy landscape. J. Chem. Phys. **153**, 134115. (10.1063/5.0016244) 33032418

[31] Mason DR, Rudd RE, Sutton AP. 2004 Stochastic kinetic Monte Carlo algorithms for long-range Hamiltonians. Comput. Phys. Commun. **160**, 140-157. (10.1016/j.cpc.2004.04.002)

[32] Bulatov VV, Oppelstrup T, Athenes M. 2011 A new class of accelerated kinetic Monte Carlo algorithms. Technical report, Lawrence Livermore National Lab.

[33] Athènes M, Bulatov VV. 2014 Path factorization approach to stochastic simulations. Phys. Rev. Lett. **113**, 230601. 25526107 10.1103/PhysRevLett.113.230601

[34] Trygubenko SA, Wales DJ. 2006 Kinetic analysis of discrete path sampling stationary point databases. Mol. Phys. **104**, 1497-1507. (10.1080/00268970600556659)

[35] Kannan D, Sharpe DJ, Swinburne TD, Wales DJ. 2020 Optimal dimensionality reduction of Markov chains using graph transformation. J. Chem. Phys. **153**, 244108. (10.1063/5.0025174) 33380101

[36] Carr JM, Wales DJ. 2008 Folding pathways and rates for the three-stranded beta-sheet peptide beta3s using discrete path sampling. J. Phys. Chem. B **112**, 8760-8769. (10.1021/jp801777p) 18588333

[37] Manhart M, Kion-Crosby W, Morozov AV. 2015 Path statistics, memory, and coarse-graining of continuous-time random walks on networks. J. Chem. Phys. **143**, 214106. (10.1063/1.4935968) 26646868 PMC4703372

[38] West AMA, Elber R, Shalloway D. 2007 Extending molecular dynamics time scales with milestoning: example of complex kinetics in a solvated peptide. J. Chem. Phys. **126**, 145104. (10.1063/1.2716389) 17444753

[39] MacKay RS. 2022 Persistence of spectral projections for stochastic operators on large tensor products. (http://arxiv.org/abs/2204.06419v1).

[40] Novotny MA, Wheeler SM. 2003 MCAMC: an advanced algorithm for kinetic Monte Carlo simulations from magnetization switching to protein folding. In *Computer Simulations of Surfaces and Interfaces*, pp. 225–235. Springer Netherlands.

[41] Novotny MA. 1995 Monte Carlo algorithms with absorbing Markov chains: Fast local algorithms for slow dynamics. Phys. Rev. Lett. **74**, 1-5. (10.1103/PhysRevLett.74.1) 10057684

[42] Puchala B, Falk ML, Garikipati K. 2010 An energy basin finding algorithm for kinetic Monte Carlo acceleration. J. Chem. Phys. **132**, 134104. (10.1063/1.3369627) 20387918

[43] Sharpe DJ, Wales DJ. 2020 Efficient and exact sampling of transition path ensembles on Markovian networks. J. Chem. Phys. **153**, 024121. (10.1063/5.0012128) 32668926

[44] Athènes M, Kaur S, Adjanor G, Vanacker T, Jourdan T. 2019 Elastodiffusion and cluster mobilities using kinetic Monte Carlo simulations: fast first-passage algorithms for reversible diffusion processes. Phys. Rev. Mater. **3**, 103802.

[45] Wales DJ. 2009 Calculating rate constants and committor probabilities for transition networks by graph transformation. J. Chem. Phys. **130**, 204111. (10.1063/1.3133782) 19485441

[46] Bobbio A, Trivedi KS. 1986 An aggregation technique for the transient analysis of stiff Markov chains. IEEE Trans. Comput. **35**, 803-814. (10.1109/TC.1986.1676840)

[47] Sonin I. 1999 The state reduction and related algorithms and their applications to the study of Markov chains, graph theory, and the optimal stopping problem. Adv. Math. **145**, 159-188. (10.1006/aima.1998.1813)

[48] Grassmann WK, Taksar MI, Heyman DP. 1985 Regenerative analysis and steady state distributions for Markov chains. Oper. Res. **33**, 1107-1116. (10.1287/opre.33.5.1107)

[49] Lal R, Bhat UN. 1988 Reduced system algorithms for Markov chains. Manag. Sci. **34**, 1202-1220. (10.1287/mnsc.34.10.1202)

[50] Heyman DP. 1995 Accurate computation of the fundamental matrix of a Markov chain. SIAM J. Matrix Anal. Appl. **16**, 954-963. (10.1137/S0895479893258814)

[51] Kohlas J. 1986 Numerical computation of mean passage times and absorption probabilities in Markov and semi-Markov models. Zeit. Oper. Res. **30**, 197-207.

[52] Heyman DP, Reeves A. 1989 Numerical solution of linear equations arising in Markov chain models. ORSA J. Comp. **1**, 52-60. (10.1287/ijoc.1.1.52)

[53] Pigolotti S, Vulpiani A. 2008 Coarse graining of master equations with fast and slow states. J. Chem. Phys. **128**, 154114. (10.1063/1.2907242) 18433197

[54] E W, Liu D, Vanden-Eijnden E. 2005 Nested stochastic simulation algorithm for chemical kinetic systems with disparate rates. J. Chem. Phys. **123**, 194107. 16321076 10.1063/1.2109987

[55] Kopelevich DI, Panagiotopoulos AZ, Kevrekidis IG. 2005 Coarse-grained kinetic computations for rare events: application to micelle formation. J. Chem. Phys. **122**, 241703. 10.1063/1.1839174 15740299

[56] Milias-Argeitis A, Lygeros J. 2013 Steady-state simulation of metastable stochastic chemical systems. J. Chem. Phys. **138**, 184109. (10.1063/1.4804191) 23676031

[57] Gillespie DT, Hellander A, Petzold LR. 2013 Perspective: stochastic algorithms for chemical kinetics. J. Chem. Phys. **138**, 170901. (10.1063/1.4801941) 23656106 PMC3656953

[58] Meyer CD Jr. 1989 Stochastic complementation, uncoupling Markov chains, and the theory of nearly reducible systems. SIAM Rev. **31**, 240-272.

[59] Trygubenko SA, Wales DJ. 2006 Graph transformation method for calculating waiting times in Markov chains. J. Chem. Phys. **124**, 234110. (10.1063/1.2198806) 16821910

[60] MacKay RS, Robinson JD. 2018 Aggregation of Markov flows I: theory. Phil. Trans. R. Soc. A **376**, 20170232. (10.1098/rsta.2017.0232) 29555805 PMC5869541

[61] Swinburne TD, Wales DJ. 2020 Defining, calculating, and converging observables of a kinetic transition network. J. Chem. Theory Comput. **16**, 2661-2679. (10.1021/acs.jctc.9b01211) 32155072

[62] Grinstead CM, Snell JL. 1997 Introduction to probability. Providence, RI: American Mathematical Society.

[63] Wales DJ. 2022 Dynamical signatures of multifunnel energy landscapes. J. Phys. Chem. Lett. **13**, 6349-6358. (10.1021/acs.jpclett.2c01258) 35801700 PMC9289951

[64] Kemeny JG, Snell JL. 1961 Finite continuous time Markov chains. Theory Probab. Appl. **6**, 101-105. (10.1137/1106012)

[65] Kemeny JG, Snell JL. 1960 Finite Markov chains. New Jersey, NJ: Van Nostrand.

[66] Le Bris C, Lelievre T, Luskin M, Perez D. 2012 A mathematical formalization of the parallel replica dynamics. Monte Carlo Methods Appl. **18**, 119-146. (10.1515/mcma-2012-0003)

[67] Bastian M, Heymann S, Jacomy M. 2009 Gephi: An open source software for exploring and manipulating networks. In *Int. AAAI Conf. on Weblogs and Social Media*. URL https://ojs.aaai.org/index.php/ICWSM/article/view/13937. 10.1371/journal.pone.0098679 PMC4051631 24914678

[68] Barabási A, Pósfai M. 2016 Network science. Cambridge, UK: Cambridge University Press.

[69] Sharpe DJ, Wales DJ. 2019 Identifying mechanistically distinct pathways in kinetic transition networks. J. Chem. Phys. **151**, 124101. (10.1063/1.5111939) 31575205

[70] Bowman GR. 2012 Improved coarse-graining of Markov state models via explicit consideration of statistical uncertainty. J. Chem. Phys. **137**, 134111. (10.1063/1.4755751) 23039589 PMC3477182

[71] Scherer MK, Trendelkamp-Schroer B, Paul F, Pérez-Hernández G, Hoffmann M, Plattner N, Wehmeyer C, Prinz J-H, Noé F. 2015 PyEMMA 2: a software package for estimation, validation, and analysis of Markov models. J. Chem. Theory. Comput. **11**, 5525-5542. (10.1021/acs.jctc.5b00743) 26574340

[72] Becker OM, Karplus M. 1997 The topology of multidimensional potential energy surfaces: theory and application to peptide structure and kinetics. J. Chem. Phys. **106**, 1495-1517. (10.1063/1.473299)

[73] Wales DJ, Miller MA, Walsh TR. 1998 Archetypal energy landscapes. Nature **394**, 758-760. (10.1038/29487)

[74] Doye JPK, Miller MA, Wales DJ. 1999 Evolution of the potential energy surface with size for Lennard–Jones clusters. J. Chem. Phys. **111**, 8417-8428. (10.1063/1.480217)

[75] Kunz RE, Berry RS. 1995 Statistical interpretation of topographies and dynamics of multidimensional potentials. J. Chem. Phys. **103**, 1904. (10.1063/1.469714)

[76] Berry RS, Breitengraser-Kunz R. 1995 Topography and dynamics of multidimensional interatomic potential surfaces. Phys. Rev. Lett. **74**, 3951. (10.1103/PhysRevLett.74.3951) 10058375

[77] Ball KD, Berry RS, Kunz RE, Li FY, Proykova A, Wales DJ. 1996 From topographies to dynamics on multidimensional potential-energy surfaces of atomic clusters. Science **271**, 963-966. (10.1126/science.271.5251.963)

[78] Berry RS, Elmaci N, Rose JP, Vekhter B. 1997 Linking topography of its potential surface with the dynamics of folding of a protein model. Proc. Natl Acad. Sci. USA **94**, 9520. (10.1073/pnas.94.18.9520) 9275155 PMC23210

[79] Swinburne TD, Kannan D. 2020 PyGT: Graph transformation and analysis in Python. https://github.com/tomswinburne/PyGT.

[80] PATHSAMPLE: a program for generating connected stationary point databases and extracting global kinetics. www-wales.ch.cam.ac.uk/software.html.

[81] Sharpe DJ. 2020 DISCOTRESS: DIscrete State COntinuous Time Rare Event Simulation Suite. https://github.com/danieljsharpe/DISCOTRESS.

[82] Woods EJ, Kannan D, Sharpe DJ, Swinburne TD, Wales DJ. 2022 Analysing ill-conditioned Markov chains supporting data [dataset]. 10.17863/CAM.88500.

